# Quantitative Differences in Nourishment Affect Caste-Related Physiology and Development in the Paper Wasp *Polistes metricus*

**DOI:** 10.1371/journal.pone.0116199

**Published:** 2015-02-23

**Authors:** Timothy M. Judd, Peter E. A. Teal, Edgar Javier Hernandez, Talbia Choudhury, James H. Hunt

**Affiliations:** 1 Department of Biology, Southeast Missouri State University, Gape Girardeau, MO, 63701, United States of America; 2 Center for Medical, Agricultural and Veterinary Entomology, USDA-ARS, 1700 SW 23 Drive, Gainesville, FL, 32604, United States of America; 3 Department of Biology, University of Missouri Saint Louis, Saint Louis, MO, 63121, United States of America; 4 Department of Biological Sciences, North Carolina State University, Raleigh, NC, 27695, United States of America; 5 Department of Entomology, W. M. Keck Center for Behavioral Biology, North Carolina State University, Raleigh, NC, 27695, United States of America; Universidade de São Paulo, Faculdade de Filosofia Ciências e Letras de Ribeirão Preto, BRAZIL

## Abstract

The distinction between worker and reproductive castes of social insects is receiving increased attention from a developmental rather than adaptive perspective. In the wasp genus *Polistes*, colonies are founded by one or more females, and the female offspring that emerge in that colony are either non-reproducing workers or future reproductives of the following generation (gynes). A growing number of studies now indicate that workers emerge with activated reproductive physiology, whereas the future reproductive gynes do not. Low nourishment levels for larvae during the worker-rearing phase of the colony cycle and higher nourishment levels for larvae when gynes are reared are now strongly suspected of playing a major role in this difference.

Here, we present the results of a laboratory rearing experiment in which *Polistes metricus* single foundresses were held in environmental conditions with a higher level of control than in any previously published study, and the amount of protein nourishment made available to feed larvae was the only input variable. Three experimental feeding treatments were tested: restricted, unrestricted, and hand-supplemented. Analysis of multiple response variables shows that wasps reared on restricted protein nourishment, which would be the case for wasps reared in field conditions that subsequently become workers, tend toward trait values that characterize active reproductive physiology. Wasps reared on unrestricted and hand-supplemented protein, which replicates higher feeding levels for larvae in field conditions that subsequently become gynes, tend toward trait values that characterize inactive reproductive physiology. Although the experiment was not designed to test for worker behavior per se, our results further implicate activated reproductive physiology as a developmental response to low larval nourishment as a fundamental aspect of worker behavior in *Polistes*.

## Introduction

Colonies of social Hymenoptera (wasps, ants, bees) are characterized by a division of reproductive labor among females in which one or more individuals reproduce and others do not. Given that worker and future reproductive (gyne) female offspring are the progeny of the same queen, the worker and gyne castes constitute an outstanding example of physiological plasticity during development [1]. Nutritional inequality during development is implicated as a variable associated with caste determination in most social insect species (reviewed in [2]). For example, larval food provisioning activates a developmental switch that leads to morphologically distinct queen and worker castes in the honey bee *Apis mellifera* [3–5] and many ant species [6] [reviewed in [7]]. A role for nutrition has also been implicated in regulating the development of reproductive castes of termites [8–10] and social aphids [11]. In all of these cases, immatures of worker castes are fed poorly relative to immatures of reproductive castes, resulting in different morphology and behavior. A role for differential larval nourishment is strongly implicated as fundamentally underlying the worker-reproductive difference in social Vespidae (reviewed in [12]).

Primitively social insects such as the paper wasp genus *Polistes* lack discrete morphological castes, and caste-related behaviors are flexible in adults [13,14]. Despite the absence of discrete morphological castes, *Polistes* species in seasonal environments have four categories of females that are easily discriminated on the basis of behavior and phase of an annual life cycle: foundresses, queens, workers, and gynes. At the start of an annual cycle, foundresses initiate nests, lay eggs, and care for their initial brood. In nests established by a single foundress, the foundress is *de facto* a solitary wasp that performs all aspects of solitary life: nest construction, oviposition, larval provisioning, and nest defense. With the emergence of her first offspring, a foundress transitions into the queen of a small society. She increases her egg production and shows a significant reduction in other aspects of maternal care. The first offspring to emerge from pupation care for brood, construct and defend the colony, and in the substantial majority of cases do not lay eggs. These are the workers. Gynes emerge following the workers and they do not lay eggs in the year in which they eclose from pupation but instead enter a phase of physiological diapause [13,15,16] and behavioral quiescence that is marked by cold hardiness [17–19] during the unfavorable season of the annual cycle. Gynes become foundresses of the following year.

The four behavioral categories of *Polistes* wasps collected in the field can be categorized on the basis of aspects of internal morphology and physiology [12,20,21]. Ovary development in the four categories in *Polistes metricus* is queens>foundresses>workers>gynes [20,22]. Abdominal lipid levels are gynes>queens & foundresses>foundresses & workers (foundresses lipid levels overlap with queen and worker levels). Hierarchical clustering of differential expression of seventeen genes in adult brains of field-collected wasps showed gynes>queens>foundresses & workers [23]. Expression levels of nine of those genes (including three in the insulin pathway) correlated positively with either lipid stores or ovary development in *Polistes metricus* [20]. In all of these contrasts, gynes are outliers in comparison to the other three categories. Contrasts of only workers and gynes show analogous differences. Gynes differ from workers in that they have abundant white abdominal fat, whereas workers have scant yellow abdominal fat [19,24]. Gyne-destined larvae accumulate larger lipid stores than worker-destined larvae [25]. Late-instar gyne-destined larvae and prepupae of field-collected wasps have higher levels of storage protein than worker-destined larvae and prepupae [26]. Gyne-destined larvae have lower levels of total protein, higher levels of potassium, and lower levels of manganese when compared to worker-destined larvae [25]. Worker-reared (putative gyne-destined) and foundress-reared (putative worker-destined) larvae of field-reared *P. metricus* differed in quantitative expression of sixteen genes, twelve of which were associated with caste and/or diapause in other insects, and the larvae also differed in abundance of nine peptides/proteins [27]. Some of the differentially-expressed genes are involved in diapause regulation in other insects, and other differentially-expressed genes and proteins are involved in the insulin signaling pathway, nutrient metabolism, and caste determination in highly social bees. Under constant laboratory conditions, field-reared gyne-destined larvae develop four days more slowly from newly-cocooned fifth instar to newly-emerged adult [15]. In all of these contrasts the difference between workers and worker-destined larvae versus gynes and gyne-destined larvae can be assigned, at least in part, to putative differences in larval nourishment. Worker-destined larvae are provisioned only by the foundress and therefore receive a low level of nourishment, whereas gyne-destined larvae are provisioned by multiple workers and receive a high level of nourishment.

Nourishment manipulation experiments using colonies of *P. metricus* in natural conditions also implicate nourishment as playing a role in aspects of individual development and subsequent reproductive differences. Supplementation of colonies with dilute honey during the pre-emergence phase yielded first-emerged offspring with higher fat levels than foundresses, whereas first-emerged offspring of un-supplemented control colonies had significantly lower fat than foundresses and first-emerged offspring of supplemented colonies [28]. Colonies supplemented with honey in the pre-emergence phase had a greater number of offspring across the full nesting season, but fewer of them remained at the nest [29]. Both of these experiments indicate a higher frequency of gynes among colonies receiving supplemental nourishment. Supplementation from nest initiation to colony decline yielded a greater number of offspring during the phase of the colony cycle when gynes would emerge [30]. In a reverse treatment, nourishment diminishment by removing nutritious larval saliva [31,32] from larvae during the pre-emergence phase of the colony cycle led to the production of few offspring overall and very few, if any, gynes [29].

All of these studies implicate larval nourishment as a factor, perhaps the primary factor, affecting caste outcomes in developing larval *Polistes*. Studies conducted in the field, however, had numerous uncontrolled variables including geographic location (Missouri, North Carolina, Illinois), source population (two study sites in Missouri were 30 km from one another, two study sites in Illinois were separated by an even greater distance), nest initiation conditions, and environmental variability. In addition, factors such as the presence and absence of nestmate larvae have been found to affect the behavior and physiology of eclosed individuals [33]. The laboratory studies cited above utilized samples collected from nests naturally established in the same field sites and therefore shared these potential problems. In addition, previous studies generally focused on only one or a few developmental variables. In order to place an explicit focus on the potential effects of environmental variability on the apparent role of larval nourishment in relation to developmental and physiological characteristics of emerged offspring, we designed an experiment to simultaneously measure as many response variables as possible using the primitively eusocial paper wasp *Polistes metricus* reared in a laboratory setting with the highest possible level of control for source population and rearing conditions. The objective was to give a higher degree of confidence than in any previously published study that variation in proteinaceous larval nourishment is the variable that fundamentally underlies the physiological traits that regulates divergence of developmental pathways that in nature lead to workers and gynes. In a restricted treatment, foundress wasps received one caterpillar every fourth day (later decreased to one every third day). In an unrestricted treatment, foundress wasps were provided with one caterpillar above the number that had been consumed since the previous day’s provisioning. In a supplemented treatment, foundress wasps were provisioned in the same manner as the unrestricted treatment, then in the evening each 5^th^-instar larva was additionally provisioned by hand using minced caterpillars and fine-point forceps. Supplemental feeding continued until a larva refused to eat further.

The hypothesis being tested was that low levels of proteinaceous nourishment during larval development would lead to developmental characteristics of workers and adults with physiological characteristics of active reproduction, whereas high levels of nourishment during larval development lead to developmental characteristics of gynes and adult physiological characteristics of inactive reproduction. In order to expose any response variables that might be subtle, the restricted and hand supplemented feeding conditions were set at extremes from one another. The unrestricted feeding treatment fell between the exaggerated treatments. Predicted traits of restricted nourishment wasps were: shorter pupal development time, smaller adult size, low abdominal fat, low hemolymph protein, and post-emergence ovary development. Predictions for the supplemented wasps were the opposite: longer pupal development time, larger adult size, high abdominal fat, high hemolymph protein, and no post-emergence ovary development. There were no specific predictions for outcomes of the unrestricted treatment beyond a tendency toward gyne-like characteristics relative to the restricted feeding treatment.

## Materials and Methods

### Wasp collection and maintenance

While standing on the roof of a house at the NC State University Honey Bee Research Facility in Raleigh, North Carolina, we collected 19 pre-foundress wasps as they exited the gable vent following overwintering within the attic. Specimen collection in this way enabled us to collect a study population with a higher probability of similar genetic background and overwintering conditions than in any previously published study of *Polistes* paper wasps. No specific permissions were required for the collection of the wasps, and the collection activities did not involve endangered or protected species.

Single wasps were placed in cages 30 cm in length, width, and height constructed of clear plastic with plastic screen on the top and two sides. An opening in the top was covered with a piece of hardboard 10 cm square with a small nest from a previous year attached at its nest stem to the underside of each hardboard. These were trimmed to seven cells ca. 0.5 cm deep, with meconia (larval feces) of the original nest occupants removed. Each seven-cell nest therefore served as a “starter” nest on which newly-captured foundress wasps could initiate construction and then expand as their own. Construction paper was the pulp source for nest construction.

Caged wasps were placed on wire racks in a growth chamber 1.22 m wide x 2.44 m long x 2.13 m high in the North Carolina State University Phytotron. The chamber contained incandescent lights on a 16L/8D cycle; fluorescent lights came on 2 h following and went off 2 h before the incandescent lights. Light intensity was 21 micromoles/sec/m^2^ in the incandescent-only morning and evening periods and 225 micromoles/sec/m^2^ during the both-lights midday. Photoperiod was constant for the full experiment, thus there was no simulated seasonality. Chamber temperature was 20°C at night and 30°C during full light, with a gradual ramp-up in the morning and ramp-down in the evening. These conditions replicated those of [34], although in a walk-in chamber rather than a cabinet-type chamber. Each cage contained small plastic Petri dishes containing sucrose (rock candy), a water source (15 ml tube plugged with cotton). Cage maintenance, conducted daily at midday, consisted of replenishing the water (deionized), replacing the cotton plug every three or four days, and providing caterpillars according to the treatment category. Each day we repositioned cages in a rotation pattern to distribute any light or temperature variation in the chamber across all colonies over the course of the experiment. Late 3^rd^- or early 4^th^-instar *Manduca sexta* larvae ca. 2.0 cm in length constituted the proteinaceous food source. In order to make the caterpillars most easily taken as prey by the wasps, head capsules of the caterpillars were quickly crushed using forceps prior to placing them into the cage. Caterpillars made moribund in this way were typically unresponsive to attack by the wasps. The majority of caterpillars uneaten before the next day’s colony maintenance were still fresh and suitable as prey.

Caterpillars for the experiment were reared in continuous culture in the North Carolina State University Insectary. The larval diet was as in [35] excluding formaldehyde as an anti-bacterial agent. The liquid mixture was poured into shallow pans that, when cooled, yielded sheets of medium on which the developing larvae fed in a rearing room maintained at 27°C. The medium for larval instars 1–3 was replaced every 2–3 days, and that for instars 4 and 5 was replaced daily. Sclerotized pupae were placed in a separate room for adult eclosion, mating, and oviposition. Eggs laid on strips of filter paper were then placed on medium in the rearing room prior to the eclosion of first instar larvae. Colony maintenance was performed daily.

### Experimental treatments

During larval provisioning, wasps knead caterpillars prior to feeding caterpillar flesh and hemolymph directly to larvae [36]. Caged wasps received one of three feeding treatments. In the restricted treatment (N = 9 colonies), wasps received one caterpillar every fourth day. After twenty days that feeding schedule was accelerated to one caterpillar every three days. In the unrestricted treatment (N = 6 colonies), wasps were provided with one caterpillar above the number that had been consumed since the previous day’s provisioning. In this treatment, one or more uneaten caterpillars in suitable condition to be used as larval provisions commonly remained the following day. In the supplemented treatment (N = 4 colonies), wasps were provisioned in the same manner as the unrestricted treatment at the midday cage maintenance, then in the evening each 5^th^-instar larva was additionally provisioned with minced caterpillar by hand using fine-tipped forceps until the larva refused to eat further.

### Specimen collection

**Fig 1:**
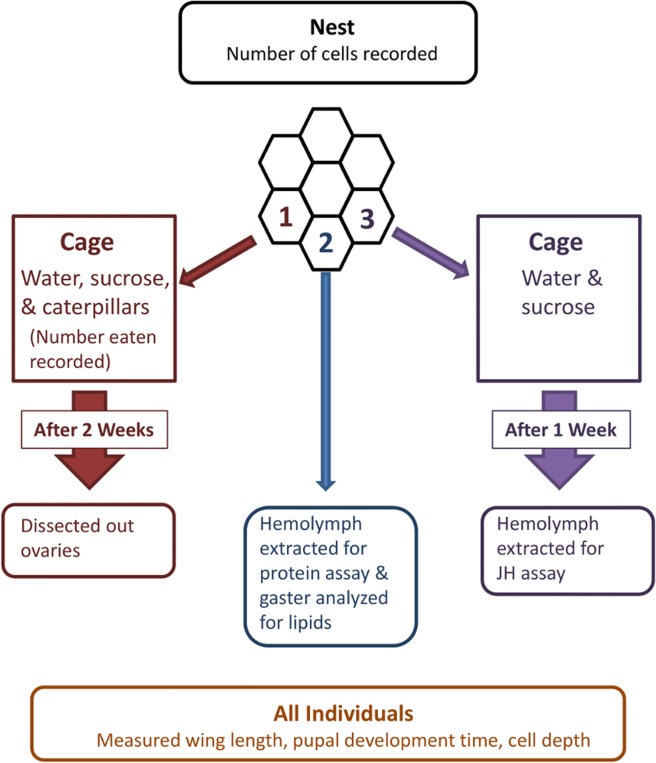
Depiction of the treatment of newly-emerged wasps during the experiment. The first emerged individual was reared in a cage for two weeks with a caterpillar supplement. After two weeks the ovaries were dissected. The second individual was frozen immediately after emergence and the levels of hemolymph protein and gaster lipids were measured. The third individual was reared for one week after eclosion and then the hemolymph was extracted for JH measurements. (The JH results were removed from the main analysis due to small sample sizes but are reported in the supplement.). This order was repeated until the end of the experiment.

The first three larvae to spin cocoons were assigned sequentially to three treatment categories for the adults that emerged from those cells (Fig. 1). The first newly emerged wasp of each treatment trio was isolated in a small cage and treated as described elsewhere [34]. Cages were kept in the same chamber as the larger cages and repositioned daily in a rotation pattern to ensure equal exposure to any variability that might exist within the chamber. Each small cage contained a small nest or nest fragment on its inner top, and each was provided with sucrose and water *ad libitum* and a late 2^nd^ or early 3^rd^-instar *Manduca sexta* caterpillar, ca. 1cm long, daily. These caterpillars had an estimated mass ca. 10% or less of those fed to foundress wasps in the large cages. Wasps were scored each day for whether or not the caterpillar had been eaten or, at least, extensively kneaded and hemolymph extracted. Wasps were held in these conditions for two weeks, at the end of which time we dissected their ovaries by cutting metasomal tergites away until only the last segment and attached organs remained. The digestive tract, Dufor’s gland, and all fat were separated from the ovaries using fine tipped forceps, then the median oviduct was separated from the terminalia. Ovaries were fixed in Dietrich’s solution in a microfuge tube for 3 days, then transferred to 70% EtOH for storage. At a later time we scored ovary development.

The second newly emerged wasp of each treatment trio was taken directly to the lab, where its hemolymph was extracted [37]. The mouthparts were sealed with ‘hot glue,’ antennae severed in mid-flagellum, and the wasp was inserted head down in a 1.5 ml microfuge tube, which was then centrifuged at 4.0 rcf (6.6 rpm) for 4 min at 4°C. Volume of hemolymph was measured using a calibrated micropipette, and buffered saline solution [38–40] (9 ml H_2_O, 0.2 mol 0.5M EDTA, 1 ml 10X PBS, and 100 mg trypsin inhibitor) was mixed with each sample at a quantity of 25% of the hemolymph volume. During processing, samples were kept on ice, handled quickly, and placed at -80° prior to processing the next specimen. At a later time we quantified protein in these samples. Following hemolymph extraction, the wasps were stored at -80°C until being analyzed for gaster lipid content.

Because juvenile hormone (JH) plays a role in caste determination in social Hymenoptera [38–42], hemolymph of the third newly emerged wasp of each treatment trio was collected for JH quantification. Unfortunately, small sample size precluded inclusion of the JH data in the statistical analyses. Details of the JH analysis and the resulting data can be found in the supplemental information (S1 Method, S1 Table).

The fourth wasp to emerge was frozen at -80°C for a genomics-based examination of nutritional effects on development that will be reported separately (Berens A, Hunt JH, Toth A in preparation). Following this, the tripartite sampling regimen (Fig. 1) was continued for as long as offspring continued to emerge.

### Bioassay procedures

Nest variables

At the conclusion of the experiment we counted the number of nest cells in which the foundress had laid an egg and the number of cells that had produced a pupa. We measured the height of pupal cocoons on the day that each was spun by using a cocoon gauge [43], recording the length in 64^ths^ of an inch using a metal machinist’s ruler. At the end of the experiment we measured the depth of each cell that had produced a pupa by placing the point of an insect pin against the meconium, grasping the pin with forceps at the margin of the paper cell to which the silk cocoon would have been measured, and quantifying the depth in 64^ths^ of an inch. Thus, total nest cell length equaled depth of the paper cell plus height of the pupal cocoon. Measurements were converted to metric units. Several cells were used to rear two larvae and had second cocoons. The join between the two cocoons can be recognized by differences in silk color (yellower on the first due to glazing) and a slight pinching-in where the second cocoon was set on the dome of the first cocoon. Cocoon height for the first cocoon of a cell is as measured. Cocoon height for second cocoons was measured as a separate height from the first cocoons. Thus, total length of a cell for those with second cocoons equaled cell depth plus the height of both cocoons.

Larval and adult variables

Each day during cage maintenance we drew a ‘nest map’ on hexagon-imprinted paper with which we recorded the number of nest cells and their contents and followed the development of each larva by its position in the nest. Larvae have five instars, and we documented daily development of each larva based on head capsule width. Because larval development time depends heavily on feeding rate, we did not quantify larval developmental time. Pupae in sealed cocoons are not fed, thus pupal development time was quantified by number of days in pupation.

We quantified adult size of all wasps in the experiment by means of a wing length measurement as in [34]. We clipped the left forewing of each wasp at its base using iris scissors, taped it to a labeled microscope slide with clear tape, and then captured digital images of the wings with an Evolution MP Color camera on an Olympus SZX12 microscope with a 0.5x objective lens and the adjustable magnification set at 10x. All images were captured in a single session with no adjustments to the equipment. Using ImageJ software, we took a linear measurement (in pixels) from the proximal closure of the C cell to the distal posterior corner of the 2Rs cell (nomenclature as in [44]). Wing wear by some wasps when in isolation precluded total wing length measurement. Measurements were blind with regard to which feeding treatment a wasp had received. We converted pixel values to mm by quantifying a mm scale photographed at the same magnification.

Ovary development

Images of ovaries of wasps in the first sample category (Fig. 1) were captured using the same protocol and equipment as for wings but with a 1.0x objective lens and 25x adjustable magnification. Ovary development was scored by independent comparisons to the same image set as used in [34] (S1 Fig.). Four ovary images represented 4 development classes: 0 = ovaries filamentous with no discernible oocytes; 1 = oocytes discernable but small in size and somewhat translucent; 2 = one or more oocytes opaque (with yolk deposition); 3 = one or more large ova present (S1 Fig.). Five non-biologists scored each image by comparison to printed copies of S1 Fig. For each ovary, we used the mean of these 5 objective scores for analyses. Scorers were blind to the objectives of the study, treatments used in the study, and the identities of the specimens. The investigator was blind to the treatment for each specimen.

Protein quantification

We used the Bradford assay [45] to estimate the amount of protein in the hemolymph of wasps in the second sample category (Fig. 1) in a manner similar to that of [25] except that the smaller amounts of available sample required the following changes. 4.0 μl of sample was combined with 80 μl of Bradford reagent and allowed to develop for 5 min. Absorbance was measured using microcuvettes in a Beckmann DU 730 Spectrophotometer. Samples were compared to 0, 0.107, 0.214, 0.428, 0.642, and 0.856 μg bovine serum standards. The standards use a 20% buffer solution similar to that used to store the hemolymph.

Lipid quantification

We removed gasters of wasps in the second sample category and determined wet mass (Fig. 1) after the hemolymph extraction. These samples were stored at -80°C prior to lipid quantification. Total lipids were estimated using the phosphovanillan assay [46] with the following changes. Gasters were homogenized in 500 μl of 1:1 chloroform methanol then centrifuged for 2 min at 14G. For each sample, a 25 μl aliquot was taken and dried. Once dried, 200 μl of concentrated sulfuric acid was added to the sample, then the sample was heated for 10 min at 100°C. 3ml of phosphovanillin reagent was added to the sample, which was allowed to develop for 30 min. The absorption of each sample was measured at 525nm on a Beckmann DU 730 Spectrophotometer. One sample exceeded the maximum measurement and was re-run using 10 μl of aliquot. The samples were compared to the absorption of 0, 18, 45, 72, and 90 μg of corn oil standards.

### Statistical analysis

All analyses showed that there was no significant difference between the unrestricted and supplemented treatments, therefore we combined the data for these two treatments and repeated the analyses using only two treatments, restricted and unrestricted. We analyzed differences between treatments for each variable using the Mann-Whitney test followed a Bonferroni table-wide correction [47]. The Mann-Whitney test was used because of small samples sizes and the ranked (ordinal) data for ovary score. In addition, we used principal component analysis to identify possible patterns of correlations among the variables and to compare morphological and physiological variables that could explain the variation among the variables scored. An advantage of PCA is that it allowed us to cluster sets of variables based on their relative variance within the experimental system. Colonies with missing data points were excluded from this analysis.

## Results

A full data set of response variables for the complete experiment can be found in S1 Table.

### Individual variable analyses

**Fig 2:**
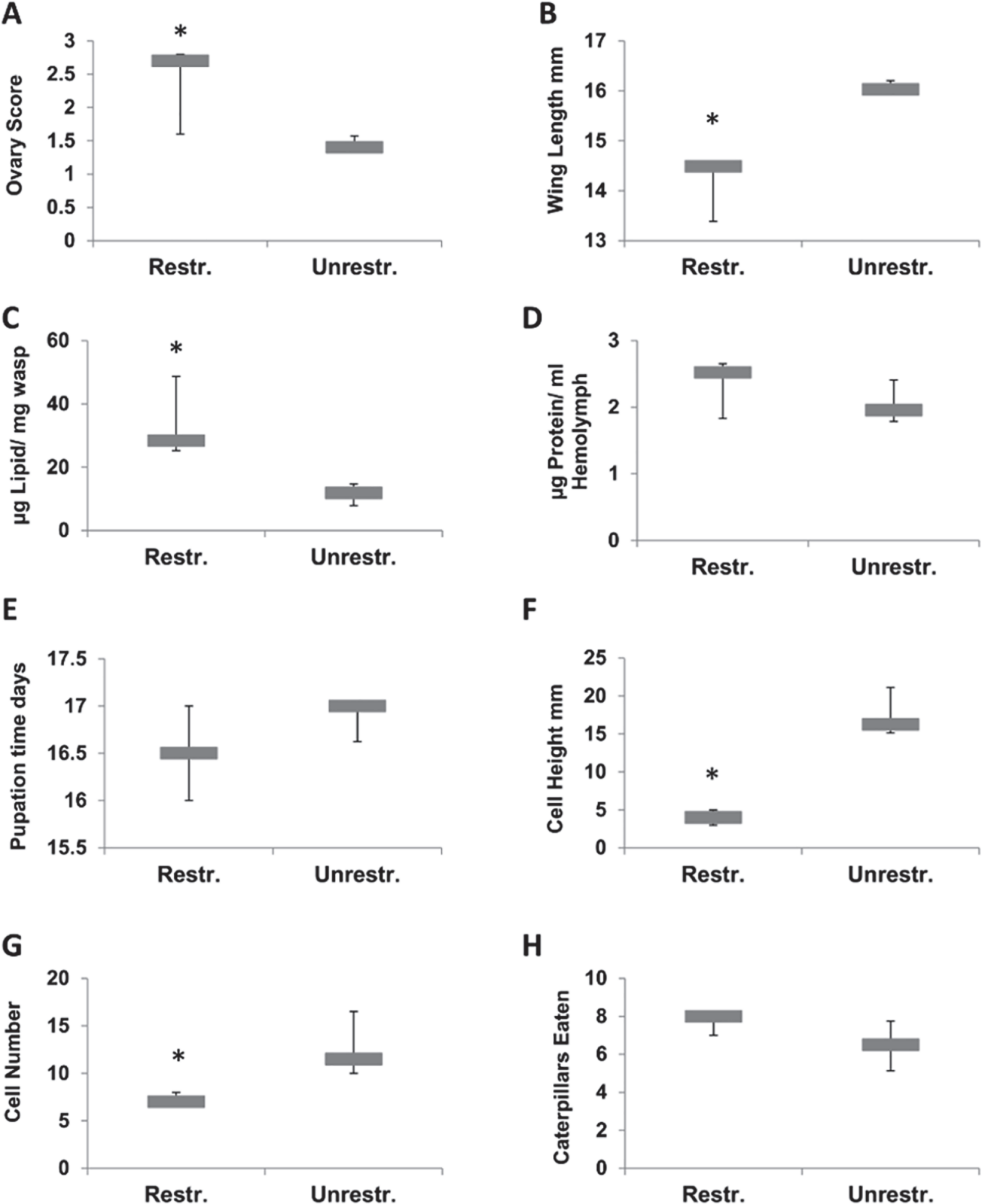
Medians (box) and quartiles of A) ovary score (N_Res_ = 8, N_Unres_ = 10 colonies), B) wing length (N_Res_ = 9, N_Unres_ = 10 colonies), C) gaster lipid levels (N_Res_ = 8, N_Unres_ = 10 colonies), D) hemolymph protein levels (N_Res_ = 7, N_Unres_ = 10 colonies), E) Number of days in pupation (N_Res_ = 9, N_Unres_ = 10 colonies), F) Pupal cocoon height (N_Res_ = 9, N_Unres_ = 10 colonies), G) number of cells in the nest (N_Res_ = 9, N_Unres_ = 10 colonies), and H) number of caterpillars eaten in two weeks by emerged wasps (N_Res_ = 9, N_Unres_ = 10 colonies). An “*” indicates cases in which the trait value in wasps from the restricted diet was significantly different from the trait values of wasps from the other two diets (Kruskal-Wallis Test, MCP). Wasps fed on *ad libium* and supplemented diets did not differ for any of the traits examined.

**Fig 3:**
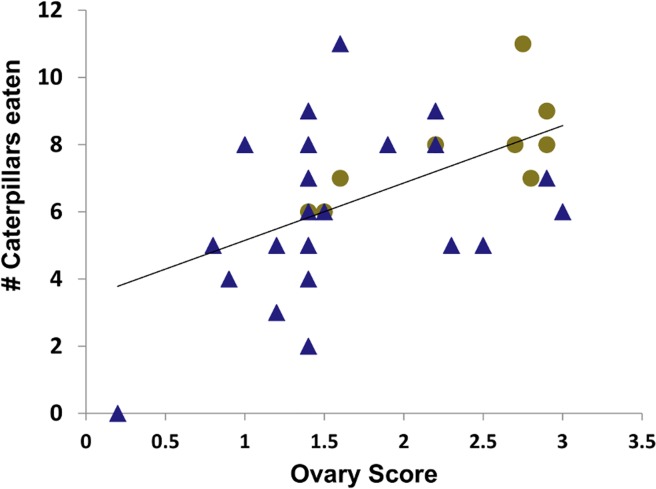
Plot of the ovary score vs. number of caterpillars eaten within the two weeks after eclosion for individual wasps reared on the restricted (gold circles, N = 9), and unrestricted (blue triangles, N = 23) diets. The trend line includes all of the data.

Of the individual variables, wing length, lipid levels, ovary score, number of nest cells, and cell height were significantly different between treatments (S2 Table). Wasps reared on the restricted diet had shorter wings, higher ovary scores, higher lipid levels, and lower cocoon heights than those in the unrestricted treatment (Fig. 2). Foundresses in the restricted treatment group produced smaller nests. The ovary score of isolated newly-emerged wasps was positively correlated with the number of caterpillars consumed during the isolation period (logistic regression, F = 10.75, p = 0.0044; Fig. 3).

### Principle components analysis

**Fig 4:**
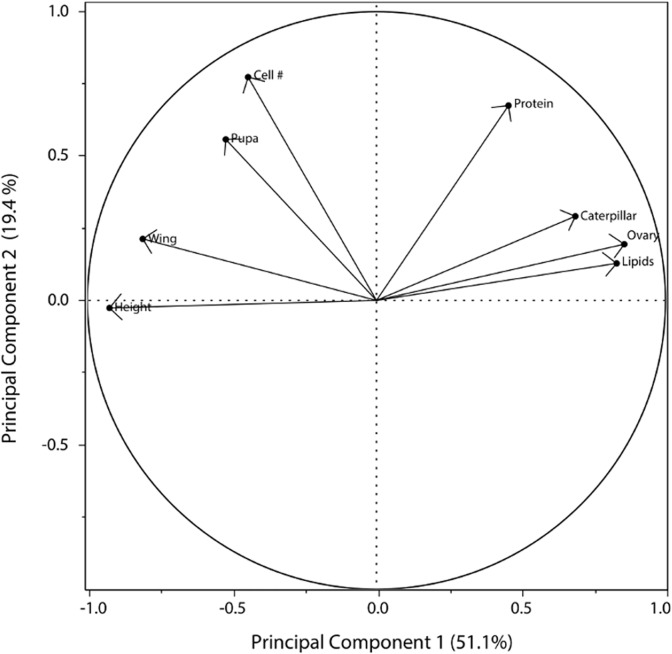
Loadings plot of PC1 versus PC2 for the two main principal component analysis obtained in the PCA analysis.

**Fig 5:**
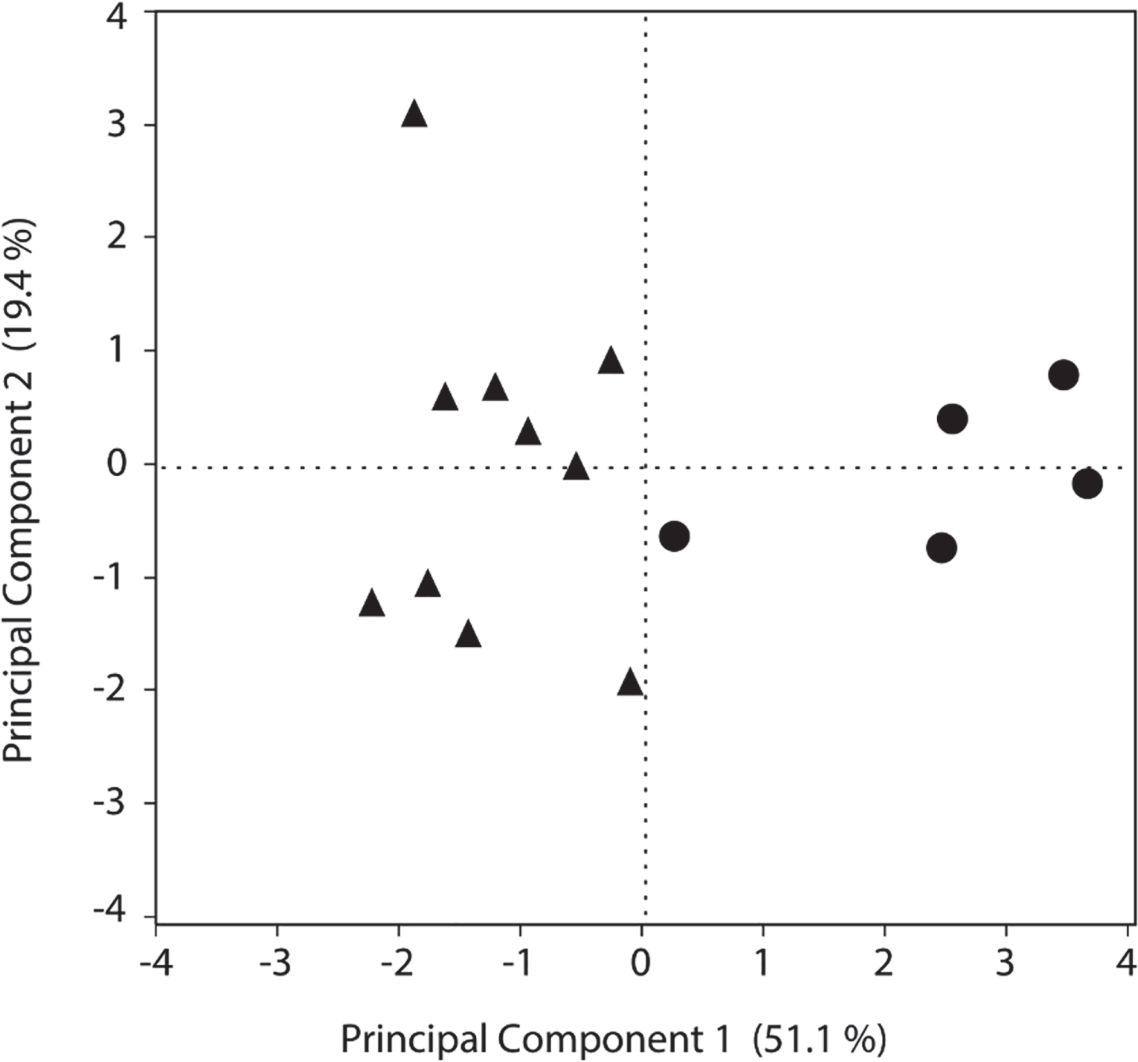
Score plot for the two main principal components obtained in the PCA analysis. Data points are separated by colonies reared on the restricted (circles), and unrestricted.

Three principal components explained more than 79.8% of the variability of the data (S3 Table). The first principal component (PC1) was negatively loaded by wing length and cell height and positively loaded by ovary score and gaster lipid levels (Fig. 4, S4 Table). The second principal component had hemolymph protein levels, nest cell number, and pupation time as the variables that explained more of the data (Fig. 4, S4 Table). The third principle component (PC3) was negatively loaded by hemolymph protein levels and gaster lipid levels and positively loaded by caterpillars eaten (S4 Table). When we clustered the contribution of the variables by the experimental unit we found that PC1 significantly separated colonies in the restricted treatment from colonies in the unrestricted treatment (F = 49.11, p<0.001, ANOVA; Fig. 5). PC2 and PC3 showed no significant differences between treatments.

## Discussion

Primitively social wasps are well-known to have caste differences in reproduction and behavior without corresponding differences in external morphology. Of the factors proposed to affect these caste differences, nutrition is considered to play a major role. Here, we controlled geographic location, source population and overwintering conditions to the fullest extent possible, light intensity, photoperiod, simulated seasonality (absent), possible variability due to cage placement in the growth chamber, temperature, nest initiation conditions, uniformity of caterpillars as larval proteinaceous nourishment, and daily cage maintenance. This level of control for non-treatment variables exceeds that of any published study. Differences in feeding level constituted the only factor that might be expected to affect outcomes to a significant degree, therefore any significant differences in outcomes have the lowest possibility of being ascribed to factors other than nutrition relative to any previous study. Results revealed four statistically significant differences between wasps reared in the restricted feeding treatment versus those reared in the unrestricted treatment. Specifically, we found that differences in nutrition between treatment levels resulted in restricted-treatment wasps emerging from pupation with higher fat levels in the gaster while being smaller in size with more developed ovaries and having lesser nest cell volume (shallower cells and lower cocoon height) than wasps reared in the unrestricted treatment. The strongest separation in the principal component analysis was between wing length and nest cell height (i.e., wasp size) versus ovary score, gaster lipid level, and caterpillars eaten in isolation prior to ovary scoring. This indicates that larger wasps tended toward lesser ovary development, lower gaster lipids, and fewer caterpillars eaten during isolation, whereas smaller wasps tended toward greater ovary development, higher gaster lipids, and more caterpillars eaten during isolation. In other words, wasps reared in the restricted feeding treatment, which are smaller than those reared in the unrestricted treatment, had characteristics likely to be associated with higher reproductive potential at the time of emergence as predicted prior to the experiment.

Wing length results match the prediction that low nourishment leads to smaller wasps. This is unsurprising. This relationship has been noted in mosquitoes [48,49], *Drosophila* [50], *Polistes fuscatus* [51,52], and in a previous study of *Polistes metricus* [18]. In *Drosophila*, there is a direct connection between diet and the wing imaginal disks. Colombani et al. [50] identified the *slif* receptor on the fat body, which appears to regulate the wing imaginal disk. There is a positive relationship with blood amino acid levels, the level of *slif*, and wing length. Thus, low amino acid levels in the hemolymph will result in shorter wings. In our study, hemolymph protein levels were not significantly different between treatments, but we did not measure free amino acid levels, which does not necessarily correlate with total protein levels [53,54].

Wasps reared in the restricted treatment had higher lipid levels in the gaster at emergence from pupation, which is counter to our prediction. In the PCA analysis, ovary score paralleled gaster lipid levels, which was also not predicted. This could have been a consequence of the high degree of dietary constraint that characterized the restricted treatment. Unlike the availability of protein, restricted treatment foundresses had unrestricted access to sugar (sucrose) and thus were able to feed carbohydrates to their larvae. Diets with a high carbohydrate to protein ratio can cause an increase in lipid levels in the insect fat body [55–58], therefore the higher ratio of carbohydrate to protein in the restricted treatment could have led to higher lipid stores. Indeed, colonies in nature of *P. metricus* that had been supplemented with honey produced first brood offspring with higher lipid levels than controls [28]. In a field study, total protein levels increased in pre-foundresses after they emerged from gyne diapause [25], which is a period of the nesting cycle in which pre-foundresses feed on flower nectar and caterpillar hemolymph as they develop their ovaries prior to nest initiation [59]. Our experimental design in which ovary development was scored only after two weeks of provisioning with caterpillars is analogous to this phase of the natural *Polistes* life cycle.

In a laboratory study of isolated wasps that emerged from nests collected in the field at dates representing the normal dates for worker and gyne emergence plus an intermediate date, Bohm [60] showed that ovarian development occurred only in *P. metricus* individuals that emerged from nests collected early in the phase of offspring production when the foundress would have been the only provisioner. A few wasps that emerged in mid-phase of offspring production showed ovary enlargement if treated topically with JH, but wasps that emerged late in the phase of offspring production showed no ovarian development even if treated topically with JH. Bohm’s ovary development data lend support to our finding that wasps reared in the restricted nourishment treatment tended toward greater ovarian development than wasps reared in the unrestricted treatment.

A particularly noteworthy finding from our study was the absence of difference in any variable between wasps in the unrestricted and hand-supplemented feeding treatments. Given that hand supplementation caused no difference between the two treatments, it is a reasonable interpretation that if a solitary foundress in nature had unrestricted access to larval provisions she would rear larvae to the full extent of their developmental potential to become gynes. What, therefore, might lead the first-emerged offspring in nature to have developmental characteristics of wasps reared experimentally on a restricted feeding regiment? There are several factors that may contribute to this. *P. metricus* is a temperate zone wasp characterized by single foundress colonies, and larvae are initially reared by a single individual during a season when daytime temperatures are cooler and nights are longer than later in the colony cycle when days are longer and warmer and larvae are being provisioned by multiple workers [16,61]. Thus, in the later stages of the colony cycle, colonies have more time each day to forage and potentially have a higher rate of prey intake due to the presence of multiple foragers. Also, during the foundress phase of the colony, after the first larva reaches its third instar, foundresses perform a behavior called “antennal drumming” that has been demonstrated to bias larvae early in a nesting cycle toward worker-like traits and behaviors in the absence of any other developmental cues [62,63]. This behavior is likely to be a “maternal manipulation” [62–64] that would amplify behavioral responses to nutritional cues at the natal nest [62] that lead first emerged offspring to remain at the natal nest, thus providing the context for energy costs of work performed by those wasps to constrain ovarian development and set the course toward nutritionally-mediated worker behavior [65]. Supporting evidence for this hypothesis comes from an epigenetic study of laboratory colonies that received simulated vibrations at a low frequency, vibrations at random frequencies, and no vibrations. Wasps emerging from nests that had been treated with low-frequency vibrations late in the colony cycle, a time when antennal drumming is not performed in nature, had worker-like gene expression related to heat shock and fat metabolism, and they performed higher rates of colony-maintenance tasks [66].

Our work shows that larval feeding level affects body size in *Polistes metricus*, with wasps from the unrestricted treatment being larger than wasps from the restricted treatment. However, size was not a determinant of reproduction in a seasonal morphometric study of 788 adult *P. metricus* in Kansas [22]. Queens were significantly smaller than fall gynes, early- and late-emerging workers were smaller than workers emerging in the mid-post-emergence season, and queens were larger than the early and late workers but equal in size to the mid-post-emergence workers. Even in light of these patterns in a natural population, our work gives evidence that the effect of nourishment on body size is less important than the effect of nourishment on reproductive physiology in the developmental divergence of workers and gynes.

Oviposition by offspring during the year in which they emerge occurs only in workers, never in gynes. Worker oviposition in the presence of the queen was observed in two large colonies of *Polistes chinensis* [67], with the ovipositing workers having foraged for about 30 days prior to the onset of oviposition [68]. In an experimental study of successive queen removals from colonies of *P. exclamans*, the replacement queen had been an active forager that, prior to becoming queen, had foraged at rates higher on average than non-replacement females [69]. Workers can found nests in mid-season, either as “satellite nests” when their natal nest is present and its colony active [70,71] or following destruction of the original nest [72,73]. Worker foundresses of *P. exclamans* satellite nests were more likely to have been foragers than wasps that remained at the natal colony [71]. Workers in orphaned colonies of *P. snelleni* were capable of mating and later producing female offspring [74]. Egg-laying workers constituted 14.7% of 273 workers from queenright colonies and 26% of 254 workers from orphaned colonies of *P. chinensis* and 8.1% of 307 workers from queen right colonies and 11.6% from 337 workers from orphaned colonies of *P. snelleni* [72]. None of these cases of worker oviposition by *Polistes* or other independent-founding Polistinae or Stenogastrinae constitute a “paradox” [74]. Neither are they an adaptive strategy based on asymmetrical patterns of relatedness in which workers that lay haploid eggs are attempting to “avoid queen control” [75] or produce their own sons to which, due to relatedness asymmetries of haplodiploidy, they are more closely related than to the sons of the queen (e.g., [76]). Instead all of these contexts in which workers lay eggs reflect the now well-supported hypothesis that the “sterile workers” have active reproductive physiology at the time of their emergence from pupation, whereas the “reproductives” (gynes) do not. Absence of oogenesis in workers of primitively social wasps without morphological castes is a reflection of behavioral dominance by the principal egg-layer (be she a foundress queen or a replacement queen) [77–80] and/or high energetic costs of work that constrain ovarian development [81]. In contexts in which dominance interactions no longer suppress oogenesis and/or the energetic costs of working are reduced or eliminated, worker phenotype wasps will undergo oogenesis, and they can and will oviposit in a diversity of contexts.

Our results give evidence drawn from a highly controlled experimental study that support the hypothesis that the divergence of worker and gyne physiology and behavior in *Polistes metricus* offspring in nature reflects a pattern of change, from low to high larval nutrition, across the brood-rearing phase of the annual colony cycle.

## Supporting Information

S1 Fig The images used to score ovary size. (DOCX)

S1 Method Juvenoid quantification. (DOCX)

S1 Table Median values per colony for all variables measured in this study. (DOCX)

S2 Table Results of the Mann-Whitney U Test followed by a Bonferroni table wide correction for all eight variables measured in the experiment. NS indicates not significant (p> 0.05).
(DOCX)

S3 Table Percent variance and cumulative percent variation of the first three principle components. (DOCX)

S4 Table Eigen values for the PCA analysis. (DOCX)
