# Supplementary figures and images for: Quantitative Differences in Nourishment Affect Caste-Related Physiology and Development in the Paper Wasp Polistes metricus
Source: PLoS One. 2015 Feb 23;10(2):e0116199. doi: 10.1371/journal.pone.0116199 (PMC4338145; doi:10.1371/journal.pone.0116199)

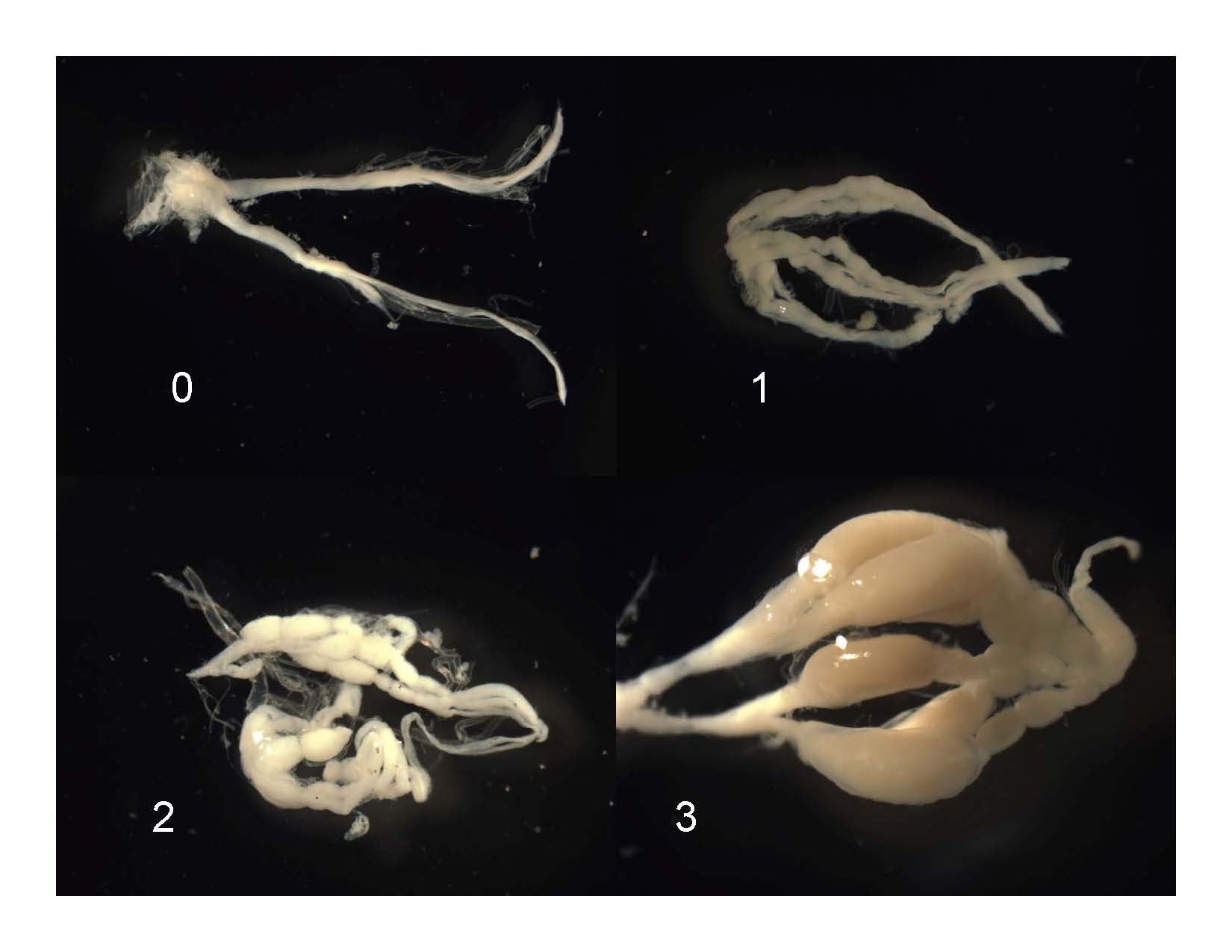


**Figure S1:** The images used to score ovary size.

Supplement: S1 Fig — (DOCX) [file pone.0116199.s001.docx]
