# Supplementary material for: Quantitative Differences in Nourishment Affect Caste-Related Physiology and Development in the Paper Wasp Polistes metricus
Source: PLoS One. 2015 Feb 23;10(2):e0116199. doi: 10.1371/journal.pone.0116199 (PMC4338145; doi:10.1371/journal.pone.0116199)
Supplement: S1 Method — (DOCX) [file pone.0116199.s002.docx]

**Method S1: Juvenoid quantification**

The third wasp of the trip was isolated in a small cage as for the 1^st^ wasp of each treatment trio, except that no caterpillars were provisioned and the wasp was isolated for only one week. This allowed the wasps to mature enough to insure the hormone levels were those of an adult and not of a juvenile. At the end of one week the hemolymph was removed, processed, and frozen as for the second-emerged wasps. At a later time we quantified juvenile hormone in these samples.

Methanolic extracts of hemolymph were prepared by addition of 500ul of pentane containing 1pg/ul each of (z)-9-tetradenecoic acid methyl ester (Z-9-C14ME) and epoxy farnesyl acetate (FA). The mixture was vortexed for 2min and centrifuged at 8000g for 10 min and the pentane layer removed to a microvial. The methanolic residual was extracted with 250ul of pentane as above 2 additional times. The combined pentane extracts were concentrated under N2 to ca 25ul prior to analysis.

Gas chromatographic-chemical ionization (isobutane reagent gas) mass spectroscopy GC-MS was conducted using an Agilent a 5975C® MSs interfaced to an Agilent 7890A® GC as in [Jones et al. [52](#_ENREF_52)]. The GC was equipped with a cool-on-column injector fitted with a 10 cm length of 0.5 mm (id) deactivated fused silica tubing connected to a 1m x 0.25 mm (id) deactivated fused silica retention gap and connected to a 30m x 0.25 mm (id, 0.25 mm coating thickness) DB5MS® analytical column. Conditions of chromatography were: initial oven and injector temperature = 30 ^o^C, 5 min; oven and injector temperatures increased at 10 ^o^C/min; final temperature = 225 ^o^C, Helium carrier gas LVF=21cm/min. The MS was operated in selective ion mode using m/z = 241 for Z-9-C14ME; m/z = 251, 219, 191 for methyl farnesoate; m/z = 267, 249 and 235 for Juvenile hormone III; and m/z = 221 and 203 for FA.
