# Supplementary material for: Quantitative Differences in Nourishment Affect Caste-Related Physiology and Development in the Paper Wasp Polistes metricus
Source: PLoS One. 2015 Feb 23;10(2):e0116199. doi: 10.1371/journal.pone.0116199 (PMC4338145; doi:10.1371/journal.pone.0116199)
Supplement: S1 Table — (DOCX) [file pone.0116199.s003.docx]

**Table S1.** Median values per colony for all variables measured in this study.

| Colony | Treat-ment | Ovary  (0-3) | Protein (µg) | JH  (pg/µl) | MF (pg/µl) | Lipids (mg/g) | Height (mm) | Pupation (days) | Cell number | Wing length (mm) | Number caterpillars eaten |
| --- | --- | --- | --- | --- | --- | --- | --- | --- | --- | --- | --- |
| **A** | Restr. | 2.2 | 2.63 |  |  |  | 10.0 | 17.5 | 7 | 14.9 | 8.0 |
| **DD** | Restr. | 2.9 | 2.52 | 0.0 | 0.041 | 23.74 | 3.0 | 16.0 | 10 | 14.5 | 9.0 |
| **FF** | Restr. | 1.5 |  | 0.0 | 0.014 | 126.13 | 4.0 | 17.0 | 8 | 14.6 | 6.0 |
| **I** | Restr. | 2.9 | 2.68 |  |  | 28.40 | -2.0 | 15.5 | 7 | 13.3 | 8.0 |
| **J** | Restr. | 1.4 | 1.98 |  |  | 26.76 | 5.0 | 16.5 | 8 | 15.6 | 6.0 |
| **L** | Restr. | 2.7 | 2.96 |  |  | 50.59 | 4.0 | 16.0 | 7 | 14.5 | 8.0 |
| **M** | Restr. | 2.8 | 1.63 | 0.0004 | 0.096 |  | 12.0 | 17.0 | 7 | 13.9 | 7.0 |
| **Q** | Restr. | 1.6 |  |  |  | 46.80 | 3.5 | 16.5 | 8 | 14.2 | 7.0 |
| **W** | Restr. | 2.75 | 1.69 |  |  | 20.85 | 0.5 | 16.0 | 6 | 14.6 | 11.0 |
| **CC** | Ad lib | 2.5 | 1.98 | 4.02 x10^-5^ | 0.055 | 11.16 | 20.0 | 17.0 | 22 | 15.8 | 5.0 |
| **EE** | Ad lib | 1.1 | 1.85 | 7.86 x10^-5^ | 0.034 | 12.58 | 15.5 | 16.5 | 10 | 16.1 | 3.5 |
| **HH** | Ad lib | 1.6 | 1.93 | 0.0044 | 0.068 | 16.82 | 15.5 | 17.0 | 11 | 15.8 | 8.0 |
| **LL** | Ad lib | 1.4 | 2.31 | 0.51 | 0.199 | 7.45 | 15.0 | 16.0 | 18 | 16.0 | 7.0 |
| **O** | Ad lib | 1.9 | 2.51 | 8.84 x10^-5^ | 0.076 | 15.26 | 13.5 | 17.0 | 12 | 16.3 | 8.0 |
| **P** | Ad lib | 1.4 | 1.76 | 3.85 x10^-5^ | 0.095 | 17.72 | 15.0 | 14.0 | 9 | 16.1 | 7.0 |
| **BB** | Suppl. | 1.3 | 2.65 | 0.0039 | 0.094 | 9.28 | 21.5 | 17.0 | 12 | 16.3 | 5.5 |
| **D** | Suppl. | 1.4 | 2.44 | 0.0 | 0.14 | 12.82 | 17.0 | 18.0 | 37 | 16.4 | 8.0 |
| **N** | Suppl. | 1.5 | 1.53 | 0.00011 | 0.085 | 5.72 | 23.0 | 17.0 | 5 | 15.0 | 6.0 |
| **U** | Suppl. | 1.05 | 1.70 | 0.00011 | 0.072 | 6.46 | 22.0 | 17.0 | 10 | 15.3 | 3.5 |
