# Supplementary material for: Quantitative Differences in Nourishment Affect Caste-Related Physiology and Development in the Paper Wasp Polistes metricus
Source: PLoS One. 2015 Feb 23;10(2):e0116199. doi: 10.1371/journal.pone.0116199 (PMC4338145; doi:10.1371/journal.pone.0116199)
Supplement: S2 Table — NS indicates not significant (p> 0.05). (DOCX) [file pone.0116199.s004.docx]

**Table S2.** Results of the Mann-Whitney U Test followed by a Bonferroni table wide correction

for all eight variables measured in the experiment. NS indicates not significant (p> 0.05).

|  | N_Restr._ | N_Unrest._ | T | p |
| --- | --- | --- | --- | --- |
| Ovary Size | 9 | 10 | 77.5 | <0.005* |
| Protein | 7 | 10 | 45 | NS |
| Lipid | 7 | 10 | 70 | <0.001* |
| Caterpillars Eaten | 9 | 10 | 66.5 | NS |
| Cocoon Height | 9 | 10 | 0 | < 0.0001* |
| Pupation Time | 9 | 10 | 31.5 | NS |
| Cell Number | 9 | 10 | 11 | <0.005* |
| Wing Length | 9 | 10 | 2 | <0.001* |

*Significant after Bonferroni Correction.
