# Supplementary material for: Quantitative Differences in Nourishment Affect Caste-Related Physiology and Development in the Paper Wasp Polistes metricus
Source: PLoS One. 2015 Feb 23;10(2):e0116199. doi: 10.1371/journal.pone.0116199 (PMC4338145; doi:10.1371/journal.pone.0116199)
Supplement: S3 Table — (DOCX) [file pone.0116199.s005.docx]

**Table S3.** Percent variance and cumulative percent

variation of the first three principle components

|  | **% Variance** | **Cumulative %** |
| --- | --- | --- |
| **PC1** | 51.05 | 51.05 |
| **PC2** | 19.36 | 70.42 |
| **PC3** | 9.43 | 79.85 |
