# Supplementary material for: Quantitative Differences in Nourishment Affect Caste-Related Physiology and Development in the Paper Wasp Polistes metricus
Source: PLoS One. 2015 Feb 23;10(2):e0116199. doi: 10.1371/journal.pone.0116199 (PMC4338145; doi:10.1371/journal.pone.0116199)
Supplement: S4 Table — (DOCX) [file pone.0116199.s006.docx]

**Table S4.** Eigen values for the PCA analysis.

|  | PC1 | PC2 | PC3 |
| --- | --- | --- | --- |
| Ovary | 0.857346 | 0.194637 | 0.227206 |
| Protein | 0.456816 | 0.674709 | -0.49658 |
| Lipids | 0.831017 | 0.128343 | -0.36494 |
| Caterpillars eaten | 0.688124 | 0.291485 | 0.512824 |
| Height of cell cap | -0.92377 | -0.02607 | -0.05784 |
| Pupation Time | -0.52136 | 0.557532 | 0.141441 |
| Cell Number | -0.44426 | 0.773243 | 0.165382 |
| Wing Length | -0.80871 | 0.21294 | -0.09424 |
